# Immune Suppression in Head and Neck Cancers: A Review

**DOI:** 10.1155/2010/701657

**Published:** 2011-03-10

**Authors:** Anaëlle Duray, Stéphanie Demoulin, Pascale Hubert, Philippe Delvenne, Sven Saussez

**Affiliations:** ^1^Laboratory of Anatomy, Faculty of Medicine and Pharmacy, University of Mons, 7000 Mons, Belgium; ^2^Department of Pathology, CHU Sart-Tilman, University of Liège, 4000 Liège, Belgium; ^3^Belgian National Fund for Scientific Research (FNRS), 1000 Brussels, Belgium; ^4^Department of Oto-Rhino-Laryngology, CHU Saint-Pierre, Université Libre de Bruxelles, 1000 Brussels, Belgium

## Abstract

Head and neck squamous cell carcinomas (HNSCCs) are the sixth most common cancer in the world. Despite significant advances in the treatment modalities involving surgery, radiotherapy, and concomitant chemoradiotherapy, the 5-year survival rate remained below 50% for the past 30 years. The worse prognosis of these cancers must certainly be link to the fact that HNSCCs strongly influence the host immune system. We present a critical review of our understanding of the HNSCC escape to the antitumor immune response such as a downregulation of HLA class I and/or components of APM. Antitumor responses of HNSCC patients are compromised in the presence of functional defects or apoptosis of T-cells, both circulating and tumor-infiltrating. Langerhans cells are increased in the first steps of the carcinogenesis but decreased in invasive carcinomas. The accumulation of macrophages in the peritumoral areas seems to play a protumoral role by secreting VEGF and stimulating the neoangiogenesis.

## 1. Epidemiology, Treatment, and Prognosis

Head and neck squamous cell carcinomas (HNSCCs) remain a significant cause of morbidity worldwide, with approximately 650,000 new cases diagnosed each year [1, 2]. HNSCCs constitute a collection of diseases that, although united by location and histology, can become very different types of tumors that differ in pathogenesis, biology, sublocation and treatment and that can affect quality of life, including survival [1, 2]. HNSCC patients associated with low clinical stages (stages I and II) have similar survival rates, with a 5-year survival between 70% and 90%, independent of the sublocation [3]. In contrast, HNSCC patients with advanced clinical stages (stages III and IV) display completely different survival rates depending on the histological type of the tumor and its sublocation [3, 4]. The treatment of HNSCC patients with advanced stages of disease combines surgery, radiation oncology, medical oncology, medical imaging, and clinical pathology [1–4]. This type of collaborative medical approach was initiated as early as 1970, when Fletcher and Evers reported the first convincing evidence of the benefits of combining radiotherapy with surgery [5]. In this context, cisplatin was investigated in the treatment of HNSCC in the early 1970s, and from the late 1970s to the early 1990s, promising results were obtained with the use of various combinations of postoperative chemotherapy with radiotherapy in randomized [6] and nonrandomized studies [7]. In the early 2000s, the Radiation Therapy Oncology Group [4] and the European Organization for Research and Treatment of Cancer (EORTC) [8] conducted two randomized studies to test the relative efficacy of concurrent postoperative cisplatin administration and radiotherapy in the treatment of HNSCC. These two studies demonstrated that local control of the disease was significantly higher in the combined therapy group than in the group that received radiotherapy alone [4, 8]. Unfortunately, these combined treatments were frequently associated with adverse side effects. Although significant progress has been observed after combined treatments, a number of statements currently remain valid concerning HNSCCs: (i) almost two-thirds of HNSCC patients have advanced forms (stages III and IV) of the disease at diagnosis, (ii) 50% of the patients die of HNSCC within the two years following initial diagnosis, and (iii) every year, 5% of the patients develop additional primary tumors. Therefore, novel approaches seem to be required to provide head and neck oncologists with a more effective armamentarium against this challenging disease [9, 10].

## 2. Immune System and Cancers

**Figure 1:**
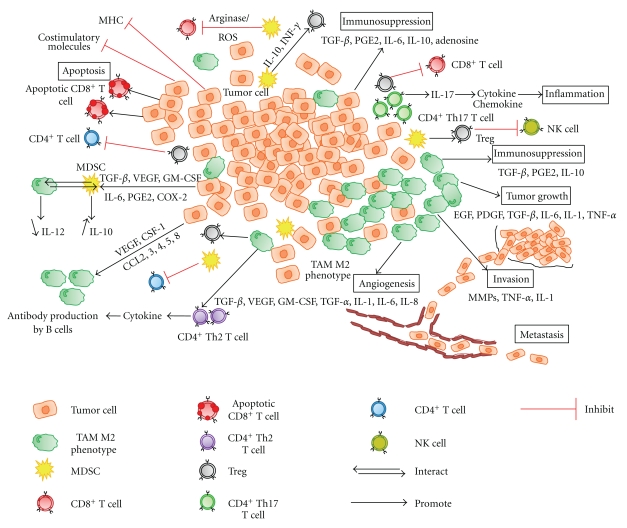
Immunosuppressive mechanisms in the tumor microenvironment: several mechanisms are developed by cancerous cells to escape to the immune system such as a loss or a reduction of the expression of MHC class 1 molecules and costimulatory molecules, the expression of FasL to induce apoptosis of tumor-infiltrating lymphocytes and the production of immunosuppressive molecules such as TGF-*β*, PGE2, IL-6, IL-10, and adenosine. Among the subpopulations of naïve CD4^+^ T cells, CD4^+^ Th17 T cells promote inflammation by secreting IL-17 whereas CD4^+^ Th2 T cells promote antibody production by B cells. Tregs promote tumor progression by inhibiting the functions of CD4^+^ and CD8^+^ T cells and NK cells. TAMs M2 phenotype induce the expression of CD4^+^ Th2 T cell and Tregs. Moreover, M2 phenotype promote growth tumor (EGF, PDGF, TGF-*β*, IL-6, IL-1, and TNF-*α*), angiogenesis (TGF-*β*, VEGF, GM-CSF, TGF-*α*, IL-1, IL-6, and IL-8), invasion (MMPs, TNF-*α*, IL-1), immunosuppression (TGF-*β*, PGE2, and IL-10) and metastasis. MDSCs induce Treg, secrete IL-10, and inhibit CD4^+^ and CD8^+^ T cells.

In the 1950s, Burnet and Thomas proposed the concept of immune surveillance of cancer. This physiological function would have the ability to recognize tumor cells as abnormal cells and to destroy them before they develop into dangerous, detectable tumors [11]. Tumor growth, invasion, and metastasis are important aspects of the tumor immune escape. The different mechanisms that are developed by tumor cells are a defect of expression of antigens on the tumor cell surface; a loss or a reduction of the expression of MHC (major histocompatibility complex) class 1 molecules, a loss of expression of costimulatory molecules, the production of immunosuppressive molecules such as transforming growth factor (TGF)-*β*, prostaglandin (PG) E2 and adenosine, or of cytokines such as interleukin (IL)-6 and IL-10, the resistance to apoptosis, and/or the expression of Fas ligand (FasL), which leads to the death of tumor-infiltrating lymphocytes (TILs) [12–15] (Figure 1).

Moreover, tumor cells recruit macrophages called tumor associated macrophages (TAMs) by secreting the colony stimulating factor (CSF-1), the chemokine ligand 2, 3, 4, 5, and 8 (CCL2, 3, 4, 5, and 8) and the vascular endothelial growth factor (VEGF) [16–18]. TAMs constitute the major inflammatory component of tumor microenvironment [19–22]. Their functions within the tumor site are various and sometimes paradoxical. Indeed, according to the environmental stimuli, macrophages present two different phenotypes. Macrophages of the M1 phenotype kill pathogens and promote the activation of cytotoxic CD8^+^ T cells and the differentiation of naïve CD4^+^ T cells into Th1 effector cells and Th17 cells [17, 18, 23]. M2 macrophages stimulate CD4^+^ Th2 cells and regulatory T cell differentiation and can promote angiogenesis and tissue remodeling [17, 18, 23] (Figure 1). Multiple studies have shown a correlation between a large number of macrophages in the tumor microenvironment and a worse prognosis. TAMs, therefore, exercise different protumor functions associated with the M2 phenotype [22, 23].

During tumor initiation, TAMs create a favorable environment for tumor growth by secreting epidermal growth factor (EGF), platelet-derived growth factor (PDGF), TGF-*β*, IL-6, IL-1, and tumor necrosis factor (TNF)-*α*. In hypoxic areas, TAMs stimulate angiogenesis (by secreting several factors, such as TGF-*β*, VEGF, granulocyte macrophage (GM)-CSF, TNF-*α*, IL-1, IL-6, and IL-8), promote tumor cell migration and invasion (via matrix metalloproteinases (MMPs), TNF-*α*, and IL-1) and induce immunosuppression (via TGF-*β*, PGE2, and IL-10). A subpopulation of TAMs, which are associated with factors such as EGF, is able to promote metastasis by guiding tumor cells in the stroma toward blood vessels, where they then escape into the circulation [16–18, 24] (Figure 1). On the other hand, other studies have shown that TAMs could also be correlated with a good prognosis. TAMs, therefore, exercise antitumor functions linked to the M1 phenotype [25–29].

In a similar way, CD4^+^ T cells can also contribute to tumor destruction or facilitate its development. Among the four subpopulations of naïve CD4^+^ T cells, type 1 CD4^+^ T cells (Th1) facilitate tumor rejection by assisting in the function of cytotoxic CD8^+^ T cells whereas type 2 CD4^+^ T cells (Th2) promote antibody production by B cells by secreting cytokines [30] (Figure 1). CD4^+^ Th17 cells, by producing IL-17, stimulate the production of cytokines and chemokines, promoting inflammation [31] (Figure 1). Several studies have shown that CD4^+^ T regulatory cells (Tregs) promote tumor progression by inhibiting the functions of T cells and natural killer (NK) cells [32, 33] (Figure 1) and that their accumulation is associated with a worse prognosis [34]. In contrast, Salama et al. have shown that the presence of Tregs is associated with a better survival rate [35].

Myeloid-derived suppressor cells (MDSCs), which are induced by VEGF, GM-CSF, TGF-*β*, IL-6, PGE2, and cyclooxygenase (COX)-2, are also implicated in tumor progression by inhibiting the actions of CD4^+^ and CD8^+^ T cells (by the production of arginase and reactive oxygen species (ROS)) [30], by inducing Tregs (through IL-10 and INF-*γ*-dependent process) [36]. They also interact with macrophages inducing a shift of the immunity towards a type 2 phenotype by increasing the secretion of IL-10 and decreasing the secretion of IL-12 [37] (Figure 1).

## 3. Immune System and Head and Neck Cancers

It appears that the origin of head and neck cancer is linked to environmental carcinogens (tobacco, alcohol) whereas tumor progression could be linked to a failure of the immune system to fight against cancer. In addition to escaping the immune system, some head and neck cancers can also corrupt the antitumor response via several mechanisms [38]. Strategies employed by head and neck cancers are varied and can target the antigen-processing machinery (APM) via the downregulation or a loss of expression of human leukocyte antigen (HLA) class I molecules and/or of other components of the APM [39, 40]. Although effective antitumor immune responses likely involve many components of the immune system, T-cells continue to be considered as the critical immune cells involved in antitumor immunity. The development of HNSCCs is strongly influenced by the host immune system [38, 41–45]. Recent evidence suggests that the antitumor responses of HNSCC patients are compromised in the presence of functional defects or apoptosis of T-cells, both circulating and tumor-infiltrating [41–45]. Tumor-derived factors or factors produced by normal cells in a local microenvironment favor tumors and disable TIL. In fact, TILs look like activated T-cells but are functionally compromised [38]. Functional assays with TILs isolated from the tumor bed have identified a number of defects, including (i) absent (or low) expression of the CD3 zeta chain (CD3*ζ*), which is the key signaling molecule in the T-cell receptor pathway [38], (ii) decreased proliferation in response to mitogens or IL-2 [38], (iii) the inability to kill tumor cell targets [44, 45], (iv) an imbalance in the cytokine profile, with the striking absence of IL-2 and/or IFN-*γ* production [46], and (v) evidence of pronounced apoptotic features in a considerable proportion of TILs [38, 47]. Moreover, immune cell dysfunction in HNSCC patients appears to extend far beyond the tumor microenvironment because both functional defects and massive lymphocyte death have also been observed in the peripheral circulation of patients with advanced HNSCC [48]. In addition, HNSCC cells that produce proinflammatory cytokines autonomously are endowed with an advantage with respect to survival and growth [49]. HNSCC cells also produce high quantities of TGF-*β*1, which reduces the expression of NK cell receptor NKG2D and CD16 and inhibits the biological functions of NK cells [50]. The induction of T-cell immunity following the vaccination of an orthotopic murine HNSCC model with a recombinant vaccinia virus expressing IL-2 induces tumor-specific CD8^+^ cytotoxic T cell (CTL) and CD4^+^ Th1-type helper T cells [51], which are targets of the cytocidal effects of galectin-1 secreted by cancer cells [52].

**Figure 2:**
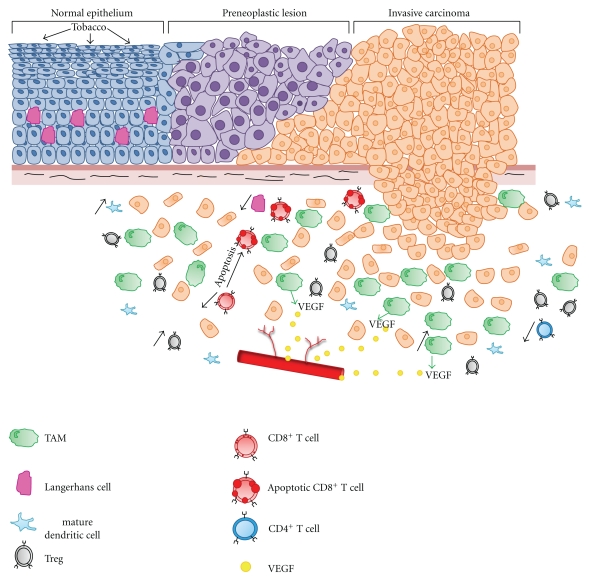
Description of immunosuppressive mechanisms during the head and neck tumor progression: in the normal epithelia of the upper aerodigestive tracts, LCs are present in the suprabasal layers. When mucosae of these areas are exposed to tobacco, the number of LCs increases whereas these cells decrease in invasive carcinomas. The mature DCs are prominent in the peritumoral area and correlated positively with the expression of VEGF. DCs are also more abundant in patients with metastasis. A higher level of TAM is observed in HNSCCs, and these cells constitute a source of VEGF which play a crucial role in angiogenesis. HNSCCs can induce the apoptosis of CD8^+^ T cells using the mitochondrial and/or Fas/FasL pathways. Tregs can induce apoptosis of CD8^+^ T cells and inhibition of the proliferation of CD4^+^ T cells.

Another mechanism employed by the tumor to escape antitumor immunity is the immunosuppressive action of Tregs. Various studies have demonstrated an increased abundance of Tregs in the TILs and of peripheral blood mononuclear cells in head and neck cancer patients [53] (Figure 2). Head and neck cancers can also directly inhibit the immune response by producing soluble mediators such as VEGF, PGE2, TGF-*β*, IL-6, and IL-10 [40, 54]. Finally, the number of TAMs seems to be correlated to the patient prognosis, suggesting possible protumoral functions of these cells in head and neck cancers [55] (Figure 2).

## 4. Disruption of the Antigen-Presenting Machinery in Head and Neck Cancers

HLAs are proteins of the MHC in humans and are present at the surface of antigen-presenting cells (APCs). T lymphocytes recognize antigens that are linked to these molecules. The APM is composed of *β* subunits of the proteolytic delta and MB1, inducible proteasome *β*-type subunits LMP2, LMP7, and LMP10, peptide transporters TAP1 and TAP2, which are essential for introducing peptides into the endoplasmic reticulum from the cytosol, and the endoplasmic reticulum chaperones calnexin, calreticulin, ERp57, and tapasin. All of these components play an important role in the generation of antigenic peptides, their translocation into the endoplasmic reticulum and loading of the *β*2-microglobulin-associated MHC class I H chain with peptides. These interactions induce the trafficking of MHC class I molecules to the cell surface and the presentation of peptides to CD8^+^ T lymphocytes [56, 57].

As mentioned previously, downregulation or loss of the expression of HLA class I molecules and/or of components of the APM is one of the strategies used by tumor cells to escape the immune system. Using immunohistochemistry, Ogino and co-authors observed a downregulation of HLA class I antigen and of most APM components in a clinical series of 63 primary laryngeal squamous cell carcinomas. Moreover, the downregulation of HLA class I antigen and of LMP2 (a component of the APM) associated with low CD8^+^ T cell infiltration were significantly associated with lower survival rates in these patients [58]. These observations were confirmed by Grandis et al., who described the loss of HLA class I protein expression in 50% of HNSCCs. This finding was also correlated with the presence of regional lymph node metastases [59]. Oral squamous cell carcinoma (OSCC)-derived gangliosides induce the downregulation of several MHC class I APM components, suggesting that this is one of the mechanisms used by the tumor to induce alterations in APM components [60].

## 5. Dendritic Cells and Head and Neck Cancers

### 5.1. Dendritic Cells Functions

Dendritic cells (DCs) are a family of specialized APCs and are essential mediators of immunity and tolerance [61, 62]. DCs are derived from the bone marrow and may have a myeloid origin (myeloid dendritic cells, MDCs) or a lymphoid origin (plasmacytoid dendritic cells, PDCs). MDCs are divided into two groups: (i) the Langerhans cells present in the epidermis and in the mucosae of the upper aerodigestive tract and (ii) the dermal/interstitial MDCs located in the dermis [63]. PDCs are found in the blood and in the T centers of lymphoid organs (thymus, tonsils, spleen, lymph nodes, etc.) [64]. In nonlymphoid tissues (peripheral tissues such as skin), DCs are immature and characterized by a high capacity for antigen capture and processing. The presence of inflammatory mediators (IL-1, TNF-*α*, and IL-12) and microbial products promotes the maturation of DCs that have lost the ability to capture antigens and have acquired an increased capacity to present antigens and to stimulate T cells. Moreover, mature DCs upregulate costimulatory molecules such as CD40, CD80, and CD86 and cytokines such as IL-1, IL-12, and TNF-*α*. Mature DCs then migrate out of nonlymphoid tissues into the blood and into secondary lymphoid organs, where they present antigens captured in peripheral tissues to T lymphocytes and stimulate T cell differentiation in effector cells (such as cytotoxic CD8^+^ T cells that are able to kill tumor cells). For these reasons, DCs can be viewed as the sentinels of the immune system [61, 65]. In contrast, immunosuppressive agents such as IL-10 and TGF-*β* convert immature DCs into tolerogenic DCs that can induce antigen-specific T-cell tolerance via several mechanisms, such as activation of Tregs, silencing of differentiated antigen-specific T cell tolerance, and differentiation of naïve CD4^+^ T cells into Tregs [66–68]. Three main immunohistochemical markers are used to detect DCs: CD1a and S-100 for immature DCs and CD83 for mature DCs.

### 5.2. Langerhans Cells and Head and Neck Cancers

Langerhans cells (LCs) are dendritic APCs located within the stratified squamous epithelium of the skin and mucosa of the upper aerodigestive tract. LCs are found in the suprabasal layers and constitute 2–8% of the intraepithelial cell content (Figure 2). Although observed in these epithelia, it is now clear that LCs are a dynamic population that migrates from the bone marrow to the stratified squamous epithelium. Regarding their roles, LCs intercept and bind new antigens detected in the squamous epithelium. Subsequently, they migrate back to the regional lymph nodes and assume the features of interdigitating dendritic cells, where they initiate a primary immune response by stimulating naïve T-lymphocytes. Later, when LCs meet recall antigens, they can present antigens to memory T-lymphocytes circulating through the extranodal skin and mucosa-associated lymphoid tissue and stimulate a secondary immune response within the mucosa [69]. Several molecules are sufficiently specific for use as LC immunohistochemical markers, such as CD1a, S100 protein and CD207.

Tobacco and alcohol consumption, which are well-established risk factors for abnormal oral mucosal changes (metaplasia and dysplasia) and oral squamous cell carcinoma, seem to be capable of stimulating mucosal LCs. Interestingly, these exposures are associated with an increased number of oral mucosal LCs (OMLCs) [69] (Figure 2). Indeed, a greater number of CD1a^+^ OMLCs has been observed in smokers at sites that are often affected by squamous cell carcinoma, such as the lips and the lateral border of the tongue [70]. Similarly, an increase in HLA-DR^+^ OMLCs in the lip has been observed [71] whereas smokeless tobacco (chewing tobacco and preparations that are absorbed by the oral or nasal mucosae (snuff)) has the opposite effect [72]. LC numbers were reportedly not associated with alcohol consumption, age, or sex, but alcohol consumption may act synergistically with tobacco use [71]. Recently, Boyle and co-authors estimated the effect of tobacco on the human oral mucosal transcriptome and demonstrated an increase of LCs in the oral mucosa of smokers [73].

The presence of S100^+^ LC in normal mucosa, premalignant and malignant lesions of the oral mucosa has been investigated by Girod et al. [74]. Their results showed a greater number of S100^+^ LCs in benign lesions than in normal mucosa. A higher LC population was also observed in the epithelium of vocal cord polyps in comparison with the normal vocal cord mucosa [75]. On the other hand, neoplastic lesions exhibited fewer S100^+^ cells than did benign lesions [74] (Figure 2). In a series of oral squamous cell carcinoma,a decrease of S100^+^ cells was shown in high-grade compared to low-grade tumors [76] (Figure 2). In laryngeal carcinomas, a strong infiltration of LCs was significantly associated with less cervical lymph node metastasis, longer disease-free survival, less locoregional recurrence and less clinical N-positivity [77]. Other studies dedicated to nasopharynx and larynx carcinomas have shown that a greater infiltration of LCs is correlated with a better prognosis [78, 79]. Moreover, the number of S100^+^ LCs decreased with the loss of tumoral differentiation [74]. These observations show that LC infiltration is prognostically important in head and neck cancers, confirming that these cells may act as important immune factors that function as APCs in the defense against HNSCCs.

### 5.3. Myeloid Dendritic Cells and Head and Neck Cancers

HNSCCs seem have a significant impact on dendritic cells (DCs). In this context, Li et al. have observed a larger number of DCs in nonmetastatic lymph nodes than in metastatic lymph nodes in a series of hypopharyngeal and laryngeal carcinomas. The immature DC marker CD1a was especially present in the cancer “nest” whereas the mature DC marker CD83 was prominent in the peritumor area [80] (Figure 2).

The relationship between the expression of VEGF, an angiogenic factor released by tumor cells, and DC infiltration, which plays an important role in immune defense against tumors, remains unclear. Therefore, several studies have analyzed the expression of VEGF isoforms in tumors. VEGF-A and VEGF-C were increased in the tumor tissue in comparison with the normal epithelium, and VEGF-D was decreased in the presence of cervical nodal metastasis. VEGF-A expression correlated with microvessel density, disease progression, a reduced number of mature DCs and an increased number of immature DCs. VEGF-A is involved in angiogenesis, tumor progression and immunosuppression [81]. Another study showed the strong expression of VEGF in oral squamous cell carcinomas from patients with regional lymph node metastasis, but in that case, the expression of VEGF was correlated inversely with the number of CD1a^+^ immature DCs and positively with the number of CD83^+^ mature DCs (Figure 2). The authors suggested that VEGF could inhibit the differentiation of CD1a^+^ immature DCs from progenitor cells and increase the levels of dysfunctional CD83^+^ mature DCs [82]. Moreover, in oral SCCs, Kikuchi and co-authors observed a greater number of S100^+^ and CD1a^+^ immature DCs in adjacent tissue and regional lymph nodes in patients without metastasis; in contrast, CD83^+^ mature DCs were more abundant in patients with metastasis [83] (Figure 2).

In lip SCCs, a higher peritumoral DC density (detected using anti-S100 antibody) was associated with a low rate of metastasis whereas a lower peritumoral DC density correlated positively with TILs. In contrast, the intratumoral DC density did not correlate with metastasis [84].

Tumor cells can modulate the expression of TLRs present on the surface of immune cells [85]. Frenzel et al. analyzed the influence of HNSCC on the TLR expression of MDCs originating from the peripheral blood. MDCs expressed all TLRs except TLR4, -9, and -7 demonstrated the strongest expression. This finding confirms that the alteration of TLR expression is an important tumor-promoting event in HNSCC progression [86]. 

HNSCCs can also influence the circulating MDC and PDC populations. So, the proportion of circulating PDCs (LIN-DR^+^123^+^) did not differ considerably in patients suffering from HNSCC compared with the healthy subjects. However, the number of circulating MDCs (LIN-DR^+^CD11c^+^) was significantly lower in patients with HNSCC. In a significant number of patients, the circulating MDC population increased after removal of the tumor, which highlights that this reduction was due to the presence of tumor and was also reversible. This deficiency in circulating MDCs could contribute to tumor immune escape in HNSCC patients [87].

### 5.4. Plasmacytoid Dendritic Cells and Head and Neck Cancers

PDCs produce large amounts of interferon (IFN)-*α* in response to viruses, and it seems that their antigen capture potential is less developed compared to other APC [88]. Hartmann et al. studied the presence and function of PDCs in HNSCC and showed that PDCs infiltrated the tumor tissue. They used oligonucleotides containing CpG motifs known as microbial stimuli for PDCs (recognized via Toll-like receptor (TLR) 9) to study the functional capacity of PDCs to produce IFN-*α*. They noticed that HNSCC PDCs decreased IFN-*α* production in response to CpG motifs. The authors hypothesized that this decreased IFN-*α* production may be due to a tumor-induced downregulation of TLR9 expression. To test this hypothesis, they determined the levels of TLRs 1–10 in PDCs from peripheral blood in the presence or absence of the supernatant from the HNSCC cell line PCI-1. In the absence of the PCI-1 supernatant, PDCs expressed high levels of TLR1, -7, and -9 and low levels of TLR6 and -10, whereas the other TLRs were at the detection limit. However, in the presence of the PCI-1 supernatant, all of these TLRs showed decreased expression levels. Therefore, the downregulation of TLR9 induced by HNSCC cells is likely one mechanism that contributes to the impaired PDC function [89]. 

PGE2 and TGF-*β* are two immunosuppressive factors found in tumor tissue. A recent study showed that TGF-*β* synergized with PGE2 inhibited IFN-*α* and tumor necrosis factor (TNF) production of TLR7- and TLR9-stimulated PDCs [90].

## 6. Macrophages and Head and Neck Cancers

Macrophages migrate from the bone marrow as immature monocytes, circulate in the bloodstream and finally migrate into tissues by extravasation to undergo differentiation into resident macrophages, including osteoclasts in the bone, alveolar macrophages in the lung, histiocytes in the connective tissue, microglia in the neural tissue, mesangial cells in the kidney, and Kupffer cells in the liver. Macrophages participate in the innate and adaptive immune systems and are critical mediators of inflammatory processes. They have several functions, including antigen presentation, target cell cytotoxicity, removal of debris and tissue remodeling, regulation of inflammation, induction of immunity, thrombosis, and various forms of endocytosis [18, 23, 91]. The main marker used in immunohistochemistry to detect macrophages of both the M1 and M2 phenotypes is CD68.

Several studies have suggested the involvement of tumor-associated macrophages (TAMs) in angiogenesis and tumor progression of HNSCCs. In a clinical series of oral carcinomas, the number of TAMs (detected by immunohistochemistry using CD68) is higher in carcinomas. A significant association between the expression of TAMs and stages of invasion, intratumoral microvessel density, and angiogenic factors such as VEGF was also observed (Figure 2) [92]. The hypothesis of the involvement of TAMs in tumoral progression was also issued during an analysis of the expression of cell cycle (cyclin E and p53) and proliferation markers (Ki67) as well as macrophage infiltration in a series of HNSCCs. In general, weak expression of Ki67, cyclin E, and p53 is associated with a better prognosis. Additionally, a direct correlation between the macrophage infiltration and the tumor proliferation index was noted, which suggested that the number of TAMs is functionally linked to tumor progression [93].

Extravascular fibrin deposits are frequently observed in the tumoral and peritumoral tissue and are involved in tumoral growth. In laryngeal and hypopharyngeal carcinomas, the accumulation of macrophages (detected using a Ki-M7 monoclonal antibody) was observed in areas of fibrin deposition, which suggests that these macrophages participate in the stabilization of intratumoral fibrin and facilitate tumor matrix generation and angiogenesis [94]. It is currently well accepted that the growth and spread of solid/malignant tumors require angiogenesis, which is described as the formation of new blood vessels in the tumor microenvironment. VEGF is a secreted endothelial cell-specific growth factor and is one of the most important factors in angiogenesis [95, 96]. Several studies have shown that apart from tumor cells, macrophages constitute a source of VEGF in carcinomas, which supports the hypothesis that macrophages play a role in tumoral formation by contributing to neovascularization [95–97] (Figure 2). Moreover, a paracrine angiogenic loop was also discovered between HNSCCs and macrophages. In fact, HNSCCs could attract macrophages by secreting MCP-1 and TGF-*β*1. Following activation, macrophages secrete VEGF and IL-8, but they also secrete TNF-*α* and IL-1, which in turn stimulate tumor cells to secrete increased levels of VEGF and IL-8 [98].

In oral SCCs, a significant correlation was observed between the presence of TAMs and the lymph node involvement and the tumor size. Hypoxia-inducible factor (HIF-1*α*) expression and TAMs can change cancer cell behavior by making them more invasive and more aggressive. The presence of tumor cell-lined vessels, HIF-1*α* expression and the high rate of TAMs could facilitate the prognosis of patients with oral squamous cell carcinoma [99]. The impact of TAMs on tumoral aggressiveness was previously studied in a series of oral cavity or oropharyngeal squamous cell carcinomas. In that study, the authors demonstrated a correlation between the aggressive behavior of HNSCCs and the level of infiltration of macrophages in the primary tumor. Indeed, the patients whose tumors showed high levels of macrophage infiltration tended to develop lymph node metastasis and to present extracapsular lymph node spread [100].

## 7. T Cells and Head and Neck Cancer

### 7.1. T Cell Functions

Immature T lymphocytes derive from stem cells of the bone marrow and mature in the thymus (primary lymphoid organ). Mature T lymphocytes leave the thymus and travel through blood and lymphoid vessels to reach secondary lymphoid organs (lymph nodes, spleen), where they are present in a naïve state [101]. In these organs, APCs can present antigens to naïve T lymphocytes. The activation of T lymphocytes requires two signals: (i) the link between MHCs from APCs and T cell receptors (TCRs) and (ii) the expression of costimulatory molecules [101]. Once activated, T lymphocytes develop into effector or memory cells. Effector cells include (i) CD4^+^ helper T cells, which facilitate B lymphocyte production of antibodies and phagocytes to destroy the ingested microbes and (ii) CD8^+^ cytotoxic T cells, which can induce cell death [101]. Helper T cells are divided into three subpopulations (Th1, Th2 and Th17), which are characterized by the secretion of various cytokines [30]. CD4^+^ T cells, or Tregs, play a critical role in the induction of tolerance to self-antigens and are divided into two main groups: naturally occurring regulatory T cells (nTregs) and peripherally induced regulatory T cells (iTregs). The iTregs include Tr1 and Th3 cells [53, 102]. Memory T lymphocytes are cells that are able to induce a rapid immune response in case of a second encounter with a previous antigen. The main immunohistochemical markers characterizing the various types of T lymphocytes are CD45RA for naïve T cells, CD45RO for memory T cells, CD69 for activated T cells, CD4 for helper T cells, CD8 for cytotoxic T cells, and CD25 and forkhead box p3 (Foxp3) for Tregs.

### 7.2. Apoptosis of T Cells in Head and Neck Cancers

Several studies have investigated the mechanisms responsible for T cell apoptosis in patients with head and neck cancer and have demonstrated that one of these mechanisms involves the Fas/FasL signaling pathway. Indeed, Gastman et al. studied the expression of FasL on the cell surface of HNSCC cells. To demonstrate that the expression of FasL on the cell surface can lead to the T cell apoptosis, they coincubated HNSCC cell lines with the Fas-sensitive Jurkat T cell line. As a result, an apoptotic signal was induced in lymphocytes, which suggests that the Fas/FasL pathway is potentially immunosuppressive [103]. They also showed that if Fas-mediated apoptosis in Jurkat cells is executed in the presence of mitochondria-specific inhibitors or synthetic caspase inhibitors, tumor-induced apoptosis is inhibited, suggesting that this phenomenon is significantly amplified by a mitochondrial loop and that tumor cells can trigger caspase-dependent apoptotic cascades in T lymphocytes [104, 105]. Once again, Hoffmann et al. showed that the Fas/FasL pathway is involved in the spontaneous apoptosis of circulating Fas^+^ T lymphocytes [48]. 

In fact, other pathways are also implicated in the T cell apoptosis. Some oral squamous cell carcinoma cell lines are also able to induce Jurkat T cell apoptosis via TRAIL and TNF-*α* [106]. Another study showed that MAGE3/6^+^FasL^+^MHC class I^+^ tumor-derived membranous vesicles isolated from the serum of patients with HNSCC induce Jurkat T cell apoptosis [107]. Moreover, Kim et al. observed that FasL^+^ membranous vesicles induced caspase-3 cleavage, cytochrome c release, loss of mitochondrial membrane potential, and reduced TCR-*ζ* chain expression and thus the mitochondrial apoptotic pathway in Jurkat and activated T cells [108].

Some pro- and antiapoptotic proteins of the mitochondrial pathway were analyzed in the lymphocytes of HNSCC patients and healthy controls. A higher level of proapoptotic Bax and antiapoptotic Bcl-XL was noted in CD8^+^ lymphocytes, as well as a higher ratio Bax/Bcl-2 in HNSCC patients compared with healthy controls. These results suggest the involvement of the mitochondrial pathway in the apoptosis of CD8^+^ T cells [109] (Figure 2).

Bcl-2 protein, an inhibitor of apoptosis, seems to be involved in the regulation of T lymphocyte apoptosis. The Bcl-2 expression in CD4^+^ and CD8^+^ T lymphocytes was significantly higher in laryngeal cancer patients than in controls. In carcinoma patients, Bcl-2 expression was also higher in CD4^+^ T cells than in CD8^+^ T cells. These results support that the Bcl-2 protein could play a role in the regulation of T lymphocyte apoptosis [110].

The hypothesis that the mechanism of Treg suppression depends on Fas/FasL-mediated apoptosis of responder cells was proposed by Strauss and colleagues. Using the blood of HNSCC patients, they showed that Tregs induced Fas-mediated apoptosis in CD8^+^ T cells (Figure 2). In contrast, CD4^+^ T cells were resistant to Fas-mediated apoptosis by Tregs but were able to induce Treg apoptosis in presence of low concentrations of IL-2 [111].

CD39 and CD73 are ectonucleotidases expressed by Tregs that convert ATP into immunosuppressive adenosine. The adenosinergic pathway in Treg-mediated suppression has also been studied in HNSCC patients. These patients demonstrated higher levels of CD39, CD73, and adenosine compared with healthy controls. This overexpression could be involved in the observed stronger effector T cell suppression [112].

Bergmann and co-authors used cell culture techniques with weak doses of IL-2, IL-10, and IL-15 to show that the tumor microenvironment generated Tr1 cells with a phenotype distinct from nTregs and that these cells abolished autologous responders proliferation via the secretion of IL-10 and TGF-*β* (Figure 2). The Tr1 cell frequency and their suppressor functions were significantly higher in patients with advanced HNSCC [102, 113].

### 7.3. T Cells and Prognosis of HNSCCs

Recently, the prognostic value of various tumor-infiltrating CD4^+^ T-cell populations (CD4^+^CD25^+^, CD4^+^CD69^+^, and CD4^+^FOXP3^+^ T cells) was determined in HNSCC patients [114]. Interestingly, a high level of CD4^+^CD69^+^ T cells was linked to a better prognosis, and CD4^+^Foxp3^+^ T cells were positively correlated with better locoregional control. In nasopharyngeal carcinomas, the density of Foxp3^+^ TILs was correlated to better overall survival and progression-free survival [115].

 Moreover, a higher density of CD4^+^CD25^+^ Tregs was also linked to a good prognosis in HNSCCs [116]. In contrast with previous studies, Strauss et al. showed that the presence of Tregs in TILs was linked to a worse prognosis in HNSCC patients. Indeed, suppression in the tumor microenvironment is mediated by a unique subset of CD4^+^CD25^high^Foxp3^+^ Tregs that produce IL-10 and TGF-*β*, exerting a more suppressive effect on proliferation [117].

The tumoral infiltration of different subpopulations of lymphocytes (CD3^+^, CD20^+^, CD43^+^, CD45^+^ RO, and CD56^+^) was assessed in laryngeal carcinomas. An increase of the CD43^+^ subpopulation was observed in the group of patients presenting lymph node metastasis. In patients with advanced carcinoma (stage IV), a correlation was established between the survival time and intensity of CD43^+^ and CD45^+^ RO lymphocyte infiltrations [118].

TCR recognizes antigens but is not able to initiate signal transduction in T lymphocytes. To achieve this, a complex must form between CD3 and the *ζ* chain linked to the TCR. The TCR-associated *ζ* chain functions as a transmembrane signaling molecule in lymphocytes [119]. Changes in the expression of the *ζ* chain of TILs are biologically significant because the absence or low expression of this chain in TILs in patients with stage III or IV HNSCC predicts a poor survival compared with patients expressing a normal *ζ* chain [120, 121]. This was confirmed by other study which demonstrated the importance of the *ζ* chain by showing that circulating CD4^+^ and CD8^+^ T cells and CD3^−^CD56^+^CD16^+^ NK cells presented lower expression of the *ζ* chain in the blood of patients with HNSCC in comparison to healthy controls. Additionally, the patients that presented a more aggressive tumor or that experienced a recurrence within the last 2 years of the study demonstrated the lowest expression levels of the *ζ* chain [122]. Reduced expression of the *ζ* chain was also noticed in laryngeal carcinomas before and after surgical treatment, and this reduction was not immediately restored after the treatment [123]. Reichert et al. studied the DC population and the expression of the *ζ* chain in TILs in a large series of 132 oral SCCs. A low density of DCs and absent or low expression of the *ζ* chain in TILs was correlated with a poor prognosis of survival and a high risk of recurrence [124].

Distel et al. tested different immunological markers using oro- and hypopharynx carcinomas in a low-risk group of 62 patients (surgery followed by radiotherapy) and in a high-risk group of 53 patients (inoperable, radiochemotherapy). The more advanced cases demonstrated higher rates of Tregs and B cells and fewer CD8^+^ T cells. In the low-risk group, a high concentration of CD20^+^ TILs was linked to a better survival rate, whereas this increase was linked to a worse prognosis in the high-risk group [125].

### 7.4. Circulating T Cells and Head and Neck Cancers

The peripheral blood of patients with tobacco-related oral SCChas shown significantly decreased CD3^+^ and CD4^+^ T cells (Figure 2). Moreover, the frequency of CD3^+^IL-4^+^ and CD8^+^IL-4^+^ T cells was significantly higher and the number of CD4^+^IL-2^+^ T cells significantly lower in these patients than in healthy controls. In late-stage cancer, the expression of IL-2 in CD4^+^ and CD8^+^ cells was also reduced [134]. IL-18 has also been assessed in patients with HNSCC, and higher levels of this cytokine seem to be produced in this type of cancer [135]. The concentrations of IL-10 were higher in patients with nodal metastasis and in T3/T4 stage tumors compared with patients without nodal metastasis and in T1/T2 stage tumors. These findings suggest that patients with advanced HNSCC exhibit a decreased Th1 immune response and an increased Th2 immune response [136].

An increase of CD4^+^CD25^+^Foxp3^+^ Tregs has been observed in the peripheral blood and in the tumor site of patients with nasopharyngeal carcinoma (Figure 2). This increase is linked to an increase in the suppressive activity of these cells on the proliferation of CD4^+^CD25^−^ T cells, which suggests the involvement of Tregs in the decreased antitumor immunity of T cells [137] (Figure 2). Increased numbers of Tregs have also been detected in HNSCCs [138]. A comparison of the numbers of CD25^+^Foxp3^+^ Tregs and CD3^+^Foxp3^+^ and CD8^+^Foxp3^+^ in TILs between oral SCCs and 15 human tumor-free tonsils again revealed an increased number of Tregs in carcinomas whereas no significant change was noted in the number of CD3^+^Foxp3^+^ and CD8^+^Foxp3^+^ in TILs [139]. Strauss et al. studied the expression of Tregs in the lymphocytes of the peripheral blood in HNSCCs. Interestingly, patients with no evident disease presented more Tregs and a stronger suppressive function than did the patients with active disease, suggesting that oncologic therapy favors the expansion of Tregs [140].

Young et al. analyzed the immune inhibitory mediators released from cancer tissues and from the immune infiltrate within the tumor in 219 HNSCCs and 64 metastatic lymph nodes. Tumor cells released substantial quantities of TGF-*β*, PGE2 and IL-10, which were associated with a decrease in CD8^+^ T cells within the tumor (Figure 2). GM-CSF, which was associated with the intratumoral presence of CD34^+^ cells, was also secreted. The authors suggested that HNSCC would evade immune suppression with reduced numbers of CD8^+^ T cells and reduced numbers or altered functions of intratumoral CD4^+^ T cells [47].

## 8. Eosinophils and Head and Neck Cancers

In HNSCCs, the function of eosinophils still remains unclear. Several studies showed that eosinophils were associated with a good prognosis [126, 127]. In fact, patients with tumour-associated tissue eosinophilia (TATE) presented higher survival in oral SCCs and less incidence of distant metastasis in head and neck cancer [126, 127]. On the other hand, some studies suggested that eosinophils were associated with a poor prognosis [128–131] or even no effect on tumor progression [132, 133]. With regard to poor prognosis, it has been shown that eosinophilic infiltration and tumor cells expressing HLA-DR antigen were correlated with an unfavorable prognosis [128]. TATE in OSCCs also reflected stromal invasion and metastasis [130, 131].

## 9. Impact of Human Papillomavirus on theImmune System of Head and Neck Cancers

It has been established that tobacco consumption and alcohol abuse are significant risk factors for the development of HNSCC but a proportion of the patients do not have these risk factors, and therefore several studies have suggested an association between the development of HNSCC and viral infection such as oncogenic (high-risk) human papilloma virus (HPV) types. The significance of hrHPV infection and its relationship with patient prognosis is still an important matter of debate, especially considering the contradictory results that are present in different studies in the literature [141, 142]. In fact, several studies have demonstrated that the presence of HPV DNA is a favourable prognostic factor with regard to recurrence and survival [143–148]. In contrast, other studies showed that patients with hrHPV positivity had a worse prognosis [142, 149, 150] or did not show a significant correlation between hrHPV infection and clinical outcomes [151–156]. A persistent HPV infection which can lead to te development of cancer requires immune tolerance and HPV developed several mechanisms for evading the host's immune system such as downregulation of IFN-*α* and TLR9, production of TGF-*β*, maintenance of low viraemia, viral gene expression and viral protein synthesis are confined to keratinocytes and the virus does not cause cell lysis and thus no inflammatory response [157]. In HNSCCs, there is an increased frequency of T cells specific for peptides derived from the oncogenic HPV E7 protein in patients whose tumors expressing HPV16 in comparison with patients whose tumors are negative for HPV or healthy volunteers. Therefore, antiviral immunity exists against E7 oncogenic protein but these T cells are unable to eliminate the tumor. So, further studies are necessary to explain this tumor's resistance [158, 159]. Williams et al. investigated whether HPV-specific immune mechanisms can result in tumor clearance. For that, they examined immune-competent and immune-incompetent mice with or without HPV. In the immune-competent mice group, one third of the HPV+ mice cleared their tumors in comparaison with none of the mice HPV−. Moreover, mice HPV+ had a significantly longer survival than mice HPV−. In the mice group lacking B- and T-cell immunity, there was no difference in growth pattern or survival between HPV+ and HPV− group. Therefore, the difference between HPV+ and HPV− mice is immune mediated. CD4+ and CD8+ T cells were found to be required to mount this immune response. They also showed that lymphocytes from mice that cleared their tumor can confer protective tumor immunity to immunoincompetent animals [160]. 

## 10. Conclusions

A better understanding of the factors that cause an immune suppression in HNSCCs might be relevant for the development of new therapeutic or prophylactic anticancer approaches. The worse prognosis of these cancers must certainly be link to the fact that HNSCCs strongly influence the host immune system. Antitumor responses of HNSCC patients are caused by the presence of functional defects or apoptosis of T-cells, both circulating and tumor-infiltrating. Langerhans cells are increased in benign tumors but decreased in invasive carcinomas. The accumulation of macrophages in the peritumoral areas seems to play a crucial role in the neoangiogenesis by secreting VEGF.
